# Biomineralized gold nanoparticles along with endophytic bacterial taxa in needles of Norway spruce (*Picea abies*)

**DOI:** 10.1186/s40793-025-00770-x

**Published:** 2025-08-28

**Authors:** Kaisa Lehosmaa, Piippa R. Wäli, Aleksi Sutinen, Janne J. Koskimäki, Maarit Middleton, Saija H.K. Ahonen, Minna Männistö, Anna Maria Pirttilä, Raimo Sutinen

**Affiliations:** 1 Ecology and Genetics Research Unit, University of Oulu, P.O. Box 3000, Oulu, FI- 90014 Finland; 2 Geological Survey of Finland, P.O. Box 77, Rovaniemi, FIN-96101 Finland; 3 Natural Resources Institute Finland, Ounasjoentie 6, Rovaniemi, 96200 Finland

**Keywords:** Au-nanoparticles, Biomineralization, EDS-FE-SEM, Amplicon sequencing, Endophytic communities, 16S rRNA

## Abstract

**Background:**

Biogeochemical techniques are used increasingly in mineral exploration to identify deposits under the sediment cover, or deep in the bedrock. Accordingly, localized biomineralized trace elements are reported in trees, but mechanisms and factors affecting the mineralization process in plant tissue are largely unknown. Localization of commercially important metals, such as gold (Au) and silver (Ag), and their vascular trafficking mechanism in trees are still poorly understood. Microorganisms play a key role in biomineralization due to their ability to influence the formation and deposition of minerals, directly or indirectly. We hypothesized a linkage between the presence of Au-nanoparticles and endophytic bacterial communities in Norway spruce needles. Therefore, we sampled 138 needles collected from 23 individual trees growing on Au mineralization in Northern Finland. We used field emission scanning electron microscopy (EDS-FE-SEM) to detect Au-nanoparticles and 16S rRNA amplicon sequencing to describe the endophytic bacterial community composition.

**Results:**

Altogether four spruce individuals, representing 17.4% of the sampled population, contained Au-nanoparticles. The Au-nanoparticles were surrounded by microbial cells encapsulated in a biofilm matrix. The bacterial richness was lower in trees with high Au concentrations, while bacterial diversity and community composition in spruce needles had no difference between trees with and without Au-nanoparticles. However, both machine learning algorithms and statistical indicator species analysis identified bacterial taxa linked with Au nanoparticle-rich needles.

**Conclusions:**

Our results suggest that Au-nanoparticles are associated with taxa such as *P3OB-42*,* Cutibacterium*, and *Corynebacterium* in Norway spruce needles. We conclude that microbes, specifically endophytic bacteria, can have a role in biomineralization processes in plants.

**Supplementary Information:**

The online version contains supplementary material available at 10.1186/s40793-025-00770-x.

## Background

Biogeochemical techniques are increasingly applied in mineral exploration to identify and prioritize exploration targets [1, 2]. In northern boreal environments, such methods are especially useful for detecting concealed Au-mineralization under the sediment cover or in the bedrock (e.g., [1]), for locating bedrock fractures, and for transporting fluids and gasses from the depth (e.g., [2]). Consequently, localized biomineralized trace elements have widely been reported in tree tissues, from both conifers and deciduous trees [3–6]. Identification of the mechanisms and factors affecting the biomineralization is important to understand the sources of bulk elemental concentrations and for generating applications in plant biogeochemical exploration.

The presence of Au-nanoparticles has earlier been reported in plant leaves in natural [2, 7–9] and artificial conditions [10]. For example, Au-nanoparticle synthesis is an emerging green method, where especially plant interaction with metallic nanoparticles has significant potential [11]. In general, heavy metal particles interfere with the plant metabolism and induce protein aggregation, leading to stress responses and decreased cell viability [12]. However, accumulation of soluble Au has only mild negative effects on vascular plants in vitro, such as growth inhibition [13, 14]. In the plant tissue, Au-nanoparticles become associated with Ca-oxalate crystals, which serve as a deduced mechanism for toxic metal compartmentalization, leading to the negation of metal toxicity [2]. The nanoparticles may also form due to reduction of Au-salts and a subsequent autocatalytic precipitation [2, 15] suggested that soluble Au is transported from soil in the transpiration stream, becomes reduced, and is then accumulated in the plant cells. However, the effect of commercially important metals, such as Au and Ag, on trees and their vascular transport mechanism is poorly understood.

In general, microorganisms play a key role in biomineralization due to their ability to influence the formation and deposition of minerals, directly or indirectly [16]. For example, bacteria have an important role in Au biomineralization in the soil due to specialized metabolic pathways [17, 18]. In their tissues, all plants harbor a diverse and rich endophytic microbiome that participates in plant metabolism and creates a functional entity, holobiont, together with the host plant [19]. Contrary to plants, accumulation of soluble Au is toxic to bacteria [20]. Therefore, we hypothesized that endophytic bacteria could have a role in translocation and biomineralization processes of trace elements, such as Au, in plant tissues.

Our aim was to investigate the presence and potential association of precipitated Au-nanoparticles with endophytic bacterial communities of Norway spruce (*Picea abies L.* Karst) needles. We used electron microscopy (FE-SEM microanalysis) to localize Au-nanoparticles and 16S rRNA gene amplicon sequencing to identify associated endophytic bacterial taxa. We focused on needles collected from spruce trees growing in the vicinity and on top of a well-documented Tiira Au-deposit, Northern Finland [21]. The mineralization is located in the Kiistala Shear Zone in connection with the Kittilä mine, which is currently the largest Au producer in Europe [21]. We expected to discover Au-nanoparticles from the trees located on the Tiira Au-deposit and to find a compositional difference in the endophytic bacterial communities along with indicator taxa between individual trees with and without Au.

## Methods

### Sampling site

**Fig. 1:**
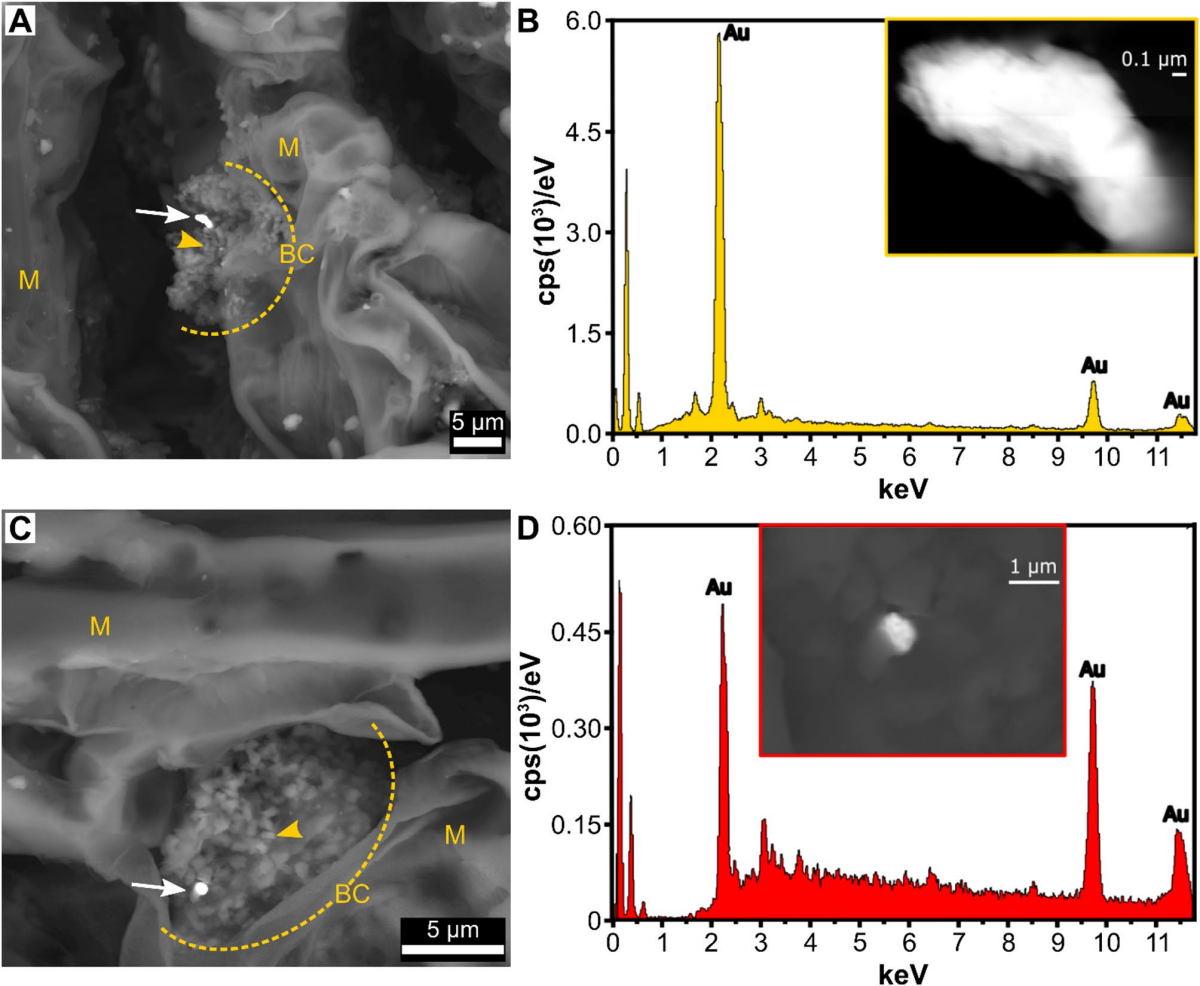
Scanning electron microscopy (SEM) for Au-nanoparticles in Norway spruce needle tissue colonised by bacteria. A detailed view of an isolated Au-nanoparticle in two different contrasts (**a-b**) with b,d) SEM with energy dispersive X-ray spectroscopy (EDS) electron-volt (eV) of Au. The acceleration voltage of 20 kV and the probe current of 0.5 nA were used to collect EDS spectra from the focused Au-nanoparticles with Cu-anode optimization. The intensity of Au-nanoparticles is seen with very bright contrast compared to the background (white arrows in a,c). The particle embedded partially into the mesophyll encapsulated in a bacterial biofilm matrix yielded much lower signal intensity compared to the particle extending outwards. Abbreviations in a,c are, BC + yellow dotted line = bacterial colony, yellow arrowhead = single bacterial cell, M = needle mesophyll. Scale bars **a, c**) 5 µm, b) 0.1 µm and **d**) 1 µm

The study was conducted on the Isokuotko deposit and its Tiira Au mineralization (25°26’ 2.73” E, 68°1’34.63” N) in the Central Lapland Greenstone Belt, Northern Finland. Iso-Kuotko deposit is known for high Au concentrations and the mineralization is structurally bound to the Kiistala Shear Zone [21] and the related Suasselkä post-glacial fault [22]. A 3D modelling based on 84 drill cores revealed an approx. 100 m by 400 m mineralization zone in several 10–100 m wide lenses of auriferous quartz-carbonate-sulfide veins, where native and refractory Au is found within arsenopyrite and pyrite. The mineralized loads are surrounded by very narrow zones of intensively altered mafic volcanic host rocks [21, 23]. A preliminary biogeochemical study of Norway spruce needles (*n* = 61 trees) on the deposit and its surroundings was conducted in 2015 (see [23]; Fig. 1) revealing Au contents of up to 4.7 µg/kg (median = 1 µg/kg, std = 1 µg/kg in ash) in spruce needles. The preliminary needle Au data was used to stratify the new sampling for the microbiome analysis. Altogether 23 mature Norway spruce trees (*P. abies L.* Karst.) were selected for the sampling of 138 needles, of which half were collected from trees with the highest Au content and another half with the lowest Au content. See Table 1 for the tree locations in relation to the 3D modelled mineralized lenses by Agnico Eagle.

**Table 1:** Concentrations of Au in dry weight of Norway Spruce needles (high > 0. 5 µg/kg, low < 0.5 µg/kg). The ore zones are interpreted from the 3D model by Agnico eagle Finland and are not exhaustive due to sparse drilling. The location of the sampled trees on the ore Lences refers to the distance of the individual tree from the detected ore Lence

Tree	Location of the sampled tree on the ore lenses	Au nanoparticles in needles detected with FE-SEM	Au concentration of needles (µg/kg)
T50	subcrop	-	<0.2 (low)
T55	depth of 50 m	-	<0.2 (low)
T57	depth of 50 m	-	2.8 (high)
T59	depth of 100–150 m	-	<0.2 (low)
T65	depth of 50 m	yes	0.7 (high)
T66	depth of 50 m	yes	<0.2 (low)
T69	depth of 100–150 m	-	0.2 (low)
T70	depth of 100–150 m	-	1.4 (high)
T74	outside ore zone (background)	-	<0.2 (low)
T77	depth of 50 m	-	<0.2 (low)
T80	outside ore zone (background)	-	<0.2 (low)
T82	outside ore zone (background)	-	<0.2 (low)
T83	outside ore zone (background)	-	<0.2 (low)
T86	outside ore zone (background)	-	<0.2 (low)
T87	outside ore zone (background)	-	0.3 (low)
T93	outside ore zone (background)	yes	0.2 (low)
T94	outside ore zone (background)	-	0.3 (low)
T95	depth of 100–150 m	yes	<0.2 (low)
T99	outside ore zone (background)	-	1.8 (high)
T101	outside ore zone (background)	-	<0.2 (low)
T102	outside ore zone (background)	-	<0.2 (low)
T103	outside ore zone (background)	-	0.3 (low)
T109	outside ore zone (background)	-	<0.2 (low)

### Sampling and sample preparation

The foliage samples were collected by choosing three spruce twigs from different sides of each tree. Twig tips of 10 cm were taken at the height of 1.5–2 m from the ground level. Each twig was packed separately and stored frozen at -24˚C. Three to four two-year-old needles were selected randomly from each twig and collected to represent two to ten needles per each tree. The needles were surface sterilized for 1 min in 70% ethanol and 20 min in 6% calcium hypochlorite, and rinsed three times with sterile water, as before [24] and cut longitudinally into two segments, one assigned to the microbiome analysis and another to the SEM analysis. The remaining needles were pooled to represent each tree in the ICP-MS analysis of elementals. One gram of dried and macerated plant pulp material was digested in HNO_3_ and aqua regia for the determination of 53 elemental concentrations (Appendix 2).

### SEM analyses

The electron microscopy analysis was conducted in a semi-quantitative manner to investigate the presence of precipitated Au-nanoparticles in the spruce needles. The needles for the SEM analysis were dried in an oven for 24 h at 60 °C. The samples were embedded in an epoxy resin, the needle segments were horizontally sectioned into 70-nm sections by a microtome, and the specimen surface was C-coated at 15-nm thickness and 2.25 g/cm^3^ density. Five to six needle segments per sampled tree were subjected to the FE-SEM microscopy. The microscopy was carried out at the GTK Research Laboratory, Espoo, Finland.

The microscopic analysis of the precipitated nanoparticles was conducted using a Field Emission Scanning Electron Microscope (FE-SEM), model JEOL JSM-7100 F Schottky, which was equipped with an EDS-spectrometer X-Max 80 mm^2^ (SDD), (Oxford Instruments, Abingdon, UK). The INCA Point ID software was used to manually analyze the obtained EDS-spectra using Cu-anode for X-ray absorbance edge optimization.

### Amplicon sequencing

Needles (n = 138) were freeze-dried and stored frozen at -24˚C for the microbiome analysis. Total DNA was extracted using NucleoSpin Plant II kit (Macherey-Nagel). The samples (half a needle per tube) were first homogenized using TissueLyserII (Qiagen) for 1.5 min at 25 Hz with two metal beads (0.5 mm), and then the NucleoSpin Plant II kit (Macherey-Nagel, Düren, Germany) protocol was followed from cell lysis step 2a (using Buffer PL1 and incubation at 65°C for 45 min). The elution was done with 30 µl of template DNA and the DNA was quantified using the NanoDrop 1000 spectrophotometer (Thermo Fisher Scientific) and Qubit dsDNA HS Assay Kit (Invitrogen). The barcoded primer pair 515F (5’-GTGCCAGCMGCCGCGGTAA-3’) and 806R (5’-GGACTACHVGGGTWTCTAAT-3’) was used to amplify the hypervariable V4 region of the 16S ribosomal RNA (rRNA) gene as previously described [25]. Additionally, synthetic peptide nucleic acid (PNA) PCR blockers, binding specifically to plant host plastid (pPNA) and mitochondrial (mPNA) 16S rRNA gene sequences, were used to suppress the PCR amplification of the host 16S templates [26]. The PCR was performed in two steps, which included controls, ZymoBIOMICS Microbial community DNA standard as the positive and molecular-grade sterile H_2_O as the negative control. First, the PCR was performed in three replicates of 50-µl reactions containing Phusion High-Fidelity DNA polymerase (Thermo Scientific) with Phusion HF buffer, 2% of DMSO, 0.4 µg/µl of BSA, 0.2 mM of dNTPs, 1.2 µM of pPNA and mPNA (PNA Bio; [26]), 0.5 µM of each primer (519F & 806R), and 3 ng of DNA. The cycling parameters consisted of an initial denaturation step of 98 °C for three minutes, followed by 30 cycles of 98 °C for 10 s, 78 °C for 10 s, 55 °C for 10 s, and 72 °C for 20 s, and lastly a final extension step of 72 °C for five minutes. The three replicate reactions from each sample were then combined and cleaned magnetically with AMPure XP (Beckman Coulter, Brea, CA, USA) and quantified with PicoGreen (Invitrogen, Carlsbad, CA, USA) by following the manufacturers’ instructions. To improve the quality of the PCR amplicons, a second PCR was done for each sample in additional 50-µl reactions with Phusion High-Fidelity DNA polymerase, Phusion GC buffer, 0.2 mM dNTPs, 0.5 µM of each primer (Ion Torrent adapter A CCATCTCATCCCTGCGTGTCTCCGACTCAG and truncated P1 CCTCTCTATGGGCAGTCGGTGAT) and 30 ng of purified PCR product. The cycling parameters consisted of an initial denaturation of 98 °C for two minutes, followed by five cycles of 98 °C for 10 s, 60 °C for 30 s and 72 °C for 30 s, and a final extension of 72 °C for five minutes. The amplicons were cleaned and quantified similar to the first PCR and then pooled into equimolar volumes and sequenced with Ion Torrent PGM system (Thermo Fisher Scientific) using Ion Torrent Hi-Q OT2 kit, Ion Torrent Hi-Q View Sequencing kit and 316 v2 chip (Thermo Fisher Scientific).

### Bioinformatics, statistical analyses, and machine learning

Single-end sequences were processed with QIIME2 (v2019.10) next-generation microbiome bioinformatics platform [27]. Short reads < 100 bp were trimmed with Cutadapt [28] and then demultiplexed according to sample-specific barcodes. The demultiplexed sequences were processed using DADA2 denoise-pyro option [29]. Altogether 3 M reads were left after trimming the primers and short reads (< 100 bp), and 1.6 M reads after denoising. The taxonomy was assigned to Exact Sequence Variants (ESVs) using naïve Bayes taxonomy classifier [30], which was first trained primer specifically with the SILVA 16S version 132 Gene Database [31]. Prior to further analyses, short (< 100 bp), as well as mitochondrial and chloroplast 16S rRNA gene sequences (covering 75% of denoised sequences) were removed, and the data was rarefied down to 1049 sequences to define the endophytic bacteriobiome. Following the removal of chloroplast and mitochondrial sequences, the data consisted of 138 samples and ~ 400 000 reads, of which 5% were classified as unassigned.

Endophytic bacterial communities were studied with respect to the SEM results (yes or no Au-nanoparticles) and Au concentration (high > 0.5 µg/kg, low < 0.5 µg/kg). Significance between the groups was tested using nonparametric permutational multivariate analysis of variance (PERMANOVA) with the *adonis* function in vegan package [32] in R statistical software [33]. PERMANOVAs were run using the Bray-Curtis similarity coefficient, and the statistical significance was estimated based on 9999 permutations. Variation in bacterial communities was visualized with nonmetric multidimensional scaling (NMDS). Species richness measured with observed taxonomic classified ESVs (later referred to as bacterial taxa) with respect to Au concentration was not normally distributed, which was tested using the Shapiro-Wilk test in R, and therefore Wilcoxon signed rank test in R was used. The richness based on SEM results was normally distributed and a parametric t-test was used in R. Indicator species analysis to determine taxa that indicate needles with Au-nanoparticles was run using *multipatt* function of indicspecies R package [34].

Machine learning analyses were conducted on the relative abundance of taxa and Au-nanoparticles using Scikit-learn [35] package in Python, and the results were visualized with Matplotlib [36]. The features that were present in less than 1% of all samples were removed before model building. The analyses were done independently using three most common tree-based machine learning models: Random Forest [37], Adaptive Boosting [38] and Extremely Randomized Trees [39]. To reduce overfitting, the machine learning models were built and validated with a leave-one-group-out 40 times repeated nested cross-validation approach, where samples from each individual spruce belonged in their own group. The parameters were tuned in the inner cross-validation loop and validated on the outer loop. To reduce the data dimensionality, each model was trained using a recurrent feature elimination process, where only the top 100 variables with the highest feature importance (MDI, mean decrease impurity) were included in the final model. Feature importance of each fold was summed and then averaged. Performance of the models were evaluated with Receiver Operating Characteristic Area Under the Curve (ROC AUC) metric, which is commonly used in machine learning model evaluation. Additionally, precision-recall area under the curve values were recorded from final models. Scikit-learn DummyClassifier models were trained to represent the random chance for each cross-validation fold.

## Results

### SEM microscopy and Au concentrations of needles

Using the FE-SEM microanalysis, we detected precipitated Au-nanoparticles in the spruce needles. Altogether, Au-nanoparticles were detected in needles of four out of 23 Norway spruce trees (Fig. 1). The Au-nanoparticle visible in Fig. 1 was localized in the mesophyll (M) extracellular matrix (EM) surrounded by extracellular material. The needle Au concentrations measured from the pooled samples varied between 0.2 and 2.8 μm/kg (in dry weight, Table 1). However, three out of four needles with Au-nanoparticles present had Au concentrations below the detection limit (Table 1). We were also able to find evidence of Ag- and As-nanoparticles, as well as nanoparticles of rare earth elements (REEs), such as Ce (Appendix 1) but concentrations of REEs, such as Ce, La, and Sc were low or under the detection limit in the needles (Appendix 2). The Ag concentration varied from 3 to 14 µg/kg (Appendix 2).

### Endophytic bacterial Microbiome of Spruce needles

We identified a total of 998 endophytic bacterial genera from the spruce needles. In terms of relative abundance, the most common bacterial phyla were *Pseudomonadota* (61%), *Bacillota* (12%) and *Actinomycetota* (12%). The phyla were equally abundant in needles with and without Au-nanoparticles. The level of dominance in the dataset was overall very high, with 91% of ESVs having a mean relative abundance below 0.1%, which signifies that only a few bacteria were highly abundant. There were six core ESVs (defined according to frequency and presence in > 80% of needles examined) in the dataset: *Cutibacterium* (96% frequency) of *Actinomycetota*, *Staphylococcus* (91% frequency) of *Bacillota*, *Sphingomonas* (88% frequency) of *Pseudomonadota*, *1174No901No12* (90% frequency), an unknown genus (86% frequency) of *Beijerinckiaceae* family, and *P3OBNo42* (81% frequency) of the *Myxococcales* order.

**Fig. 2:**
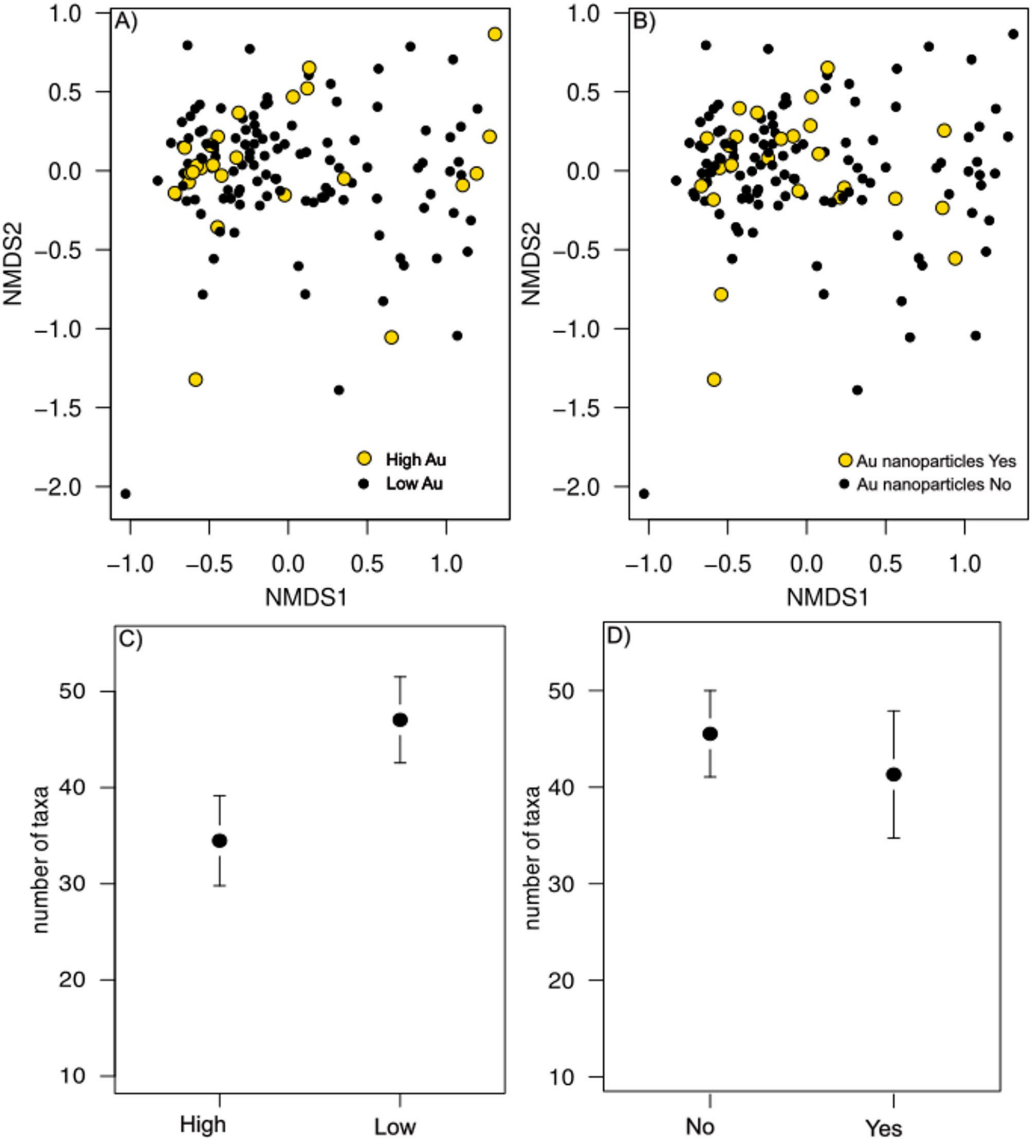
Nonmetric multidimensional scaling (NMDS) illustrating (**a**) compositional difference of endophytic bacteria between Norway spruce needles with low (black dots) and high (yellow dots) Au concentrations. (**b**) Compositional difference between the needles with Au-nanoparticles (yellow dots) and without Au-nanoparticles (black dots). Figures c-d represent means (± 95% CI) of bacterial richness between low and high Au concentrations (**c**) and **d**) between with and without Au-nanoparticles in the studied needles

The linkages between the endophytic bacterial community structure and Au were then studied based on the presence of Au-nanoparticles (FE-SEM: yes or no) or Au concentrations (high > 0.5 µg/kg, low < 0.5 µg/kg). The Au concentration (PERMANOVA, *F*_*1,136*_ = 1.44, *P* > 0.05; Fig. 2a) or the presence of Au-nanoparticles (PERMANOVA, *F*_*1,136*_= 0.67, *P* > 0.05) (Fig. 2b) did not significantly affect the bacterial community structure. However, the species richness of endophytic bacteria was significantly lower (*P* = 0.005) in needles with high Au concentration (Fig. 2c), while the species richness did not differ based on the presence of Au-nanoparticles (*P* > 0.05, Fig. 2d). Furthermore, the indicator species analyses revealed 18 bacterial taxa as indicators of Au-nanoparticles in needles (Appendix 3). Of these, the most evident were the genus *Corynebacterium* and an unknown genus of the *P3OB-42* family (*Myxococcales*).

Machine learning was further applied on the bacterial community data grouped by the presence of Au-nanoparticles (FE-SEM results). The Random Forest (RF) algorithm achieved the highest AUC values by the two metrics used, ROC AUC and PR AUC (Appendix 4; with and without Au-nanoparticle). ROC evaluates the ability to discriminate between classes across all thresholds, while PR focuses on the model’s precision and recall in imbalanced datasets. AdaBoost had significantly lower ROC AUC compared to RF (~ 0.80 vs. ~ 0.70) but had a similar PR AUC performance (0.63 vs. 0.62), revealing that PR and ROC had differences when evaluating classifier performances. ExtraTrees produced the lowest AUC values, in general, such underperforming can occur due to overfitting, sensitivity to noise, or lack of robustness, making it a less reliable option.

**Fig. 3:**
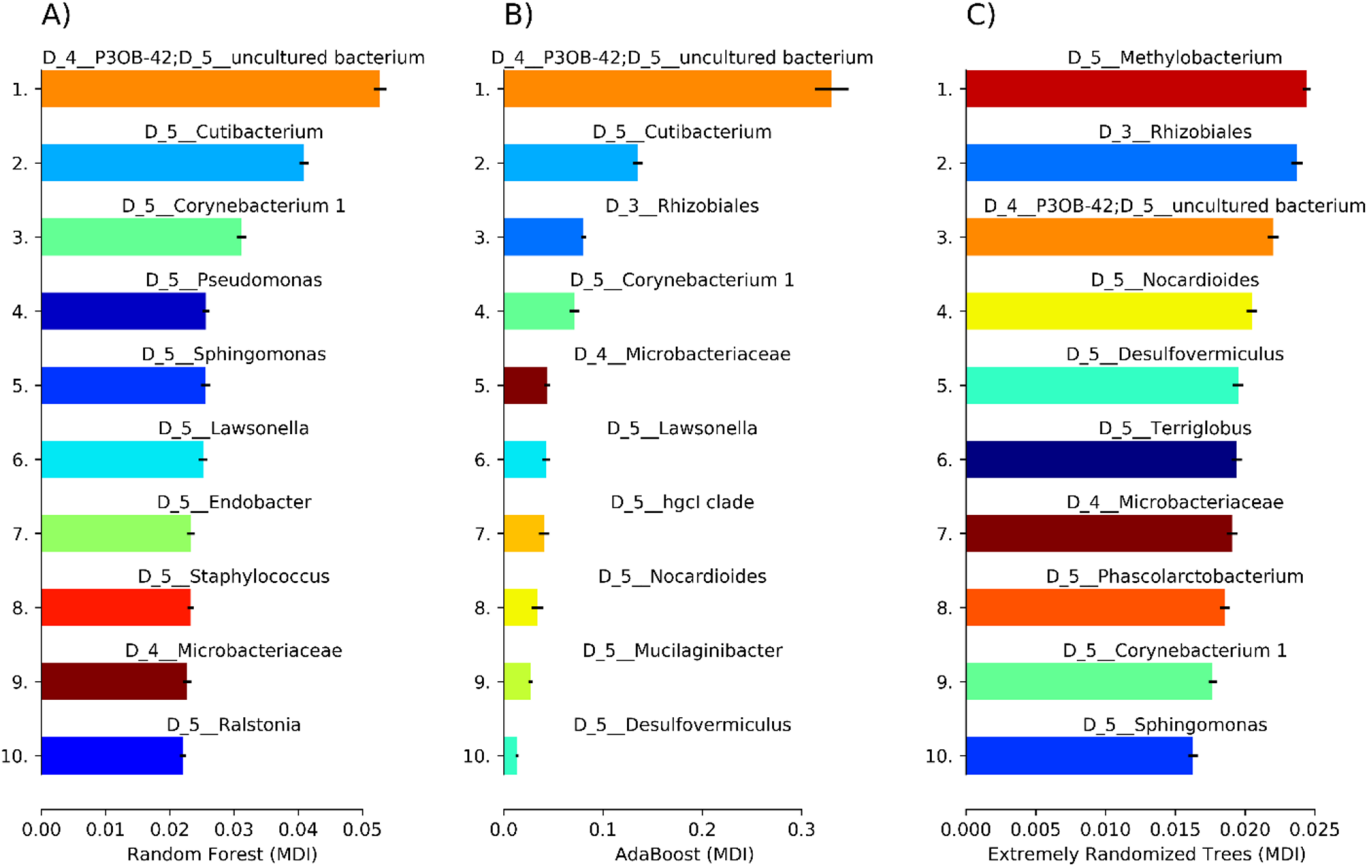
Feature importance plot for Au-nanoparticle models (MDI, mean decrease impurity) bar charts with shortened SILVA taxonomic IDs for each algorithm, (**a**) Random Forest, (**b**) AdaBoost and (**c**) ExtraTrees models. D_4 refers to bacterial family and D_5 genus level identification

The MDI values within the models and the highest variables were among the most important for separating the classes between presence or absence of Au-nanoparticles. The feature importance plots of RF (Fig. 3a) and AdaBoost (Fig. 3b) showed that the uncultured bacterium from the family *P3OB-42* was the top-performing variable in separating the groups of Au-nanoparticles in needles. The genera *Cutibacterium* and *Corynebacterium* also appeared in both models as the top-ranking variables, whereas Extratrees identified the *Methylobacterium* genus as the top-ranking variable (Fig. 3c). However, due to the low AUC values, the Extratrees was the least trustworthy algorithm.

## Discussion

Presence of metals, such as Au-nanoparticles, has been reported in plant leaves [7, 9, 10, 40, 41], but the process of biomineralization has remained unknown. Plants host numerous microbial species, endophytes, in their tissues, and we hypothesized their potential participation in the biomineralization process in plants. Our detailed analytical, microscopy and molecular biology-based studies supported by rigorous statistical and machine learning analyses provided evidence for microbial interference in accumulation of metal particles in living plant tissue.

We detected Au-nanoparticles in needles of four out of 23 Norway spruce trees, indicating that the spruce trees can take up soluble Au in natural conditions via the roots and translocate it to aerial tissues, where Au becomes precipitated. The Au-nanoparticles were detected in the intercellular matrix of spruce needle mesophyll. Because they were identified inside the living, photosynthesizing tissue of trees mainly located at the ore zone, they were likely transported from the soil. We were also able to find evidence of Ag and As nanoparticles in the spruce needles, which can be connected to the mineralogy of the orogenic Au-deposit at Tiira, where Au exists within arsenopyrite and associated trace elements [21, 23]. Additionally, rare earth elements (REEs), such as Ce, were identified in the spruce needles in a similar manner as Au-nanoparticles, although the Tiira Au ore is not especially enriched with REEs. The Ce nanoparticles were found from another set of trees, located apart from the Au-deposit site, and the concentrations of Ce within the needles were under the detection limit.

The Au-nanoparticles were covered by microbial cells embedded in a biofilm. Biofilm is a living material composed by different microbial species that generate an exopolysaccharide matrix [9], specific to plant-associated bacteria [10]. It is often found on various surfaces and constructed by endophytic microbes inside plants [42–44]. To evaluate whether an endophytic community was associated with the Au-nanoparticles, we analyzed the bacterial communities of the needles.

The bacterial endophytic communities of Norway spruce phyllosphere have not been studied earlier, and the communities were characterized by the bacterial phyla *Pseudomonadota*, *Actinomycetota*, and *Bacillota*, as well as the six core ESVs, *Cutibacterium*, *Staphylococcus*, *Sphingomonas*, *1174No901No12*, an unknown genus of *Beijerinckiaceae*, and *P3OBNo42* of the *Myxococcales* order. We discovered that the needles with high Au concentration had a lower species richness, which is consistent with earlier results that high metal concentrations reduce biodiversity of microbial communities [45, 46]. The Au concentration or the presence of Au-nanoparticles had no effect on the community structure of endophytic bacteria. This suggests that the microbes participating in the biomineralization process originated from the existing innate endophytic community.

Indeed, *Corynebacterium* and unclassified species from the genera *Cutibacterium* were associated with Au-nanoparticles in the spruce needles by indicator species analysis and the machine learning algorithms Random Forest and AdaBoost. The genus *Methylobacterium* was also an Au-associated candidate in the needles of Norway spruce by the machine learning model Extratrees. Due to their proposed role in Au-nanoparticle accumulation by two unrelated analysis methods, these bacterial taxa deserve a brief examination with respect to their association with metals and higher organisms, to further evaluate the likelihood of their involvement in metal nanoparticle formation in plants.

Recently, the members of the bacterial family *P3OB-42* have been identified in the phyllosphere of *Pinus koraiensis* [47]. The family *P3OB-42* has also been discovered in the bacterial community of *Imperata cylindrica* litter in a copper tailings dam, where Cu values of up to 487 mg/kg were reported in the soil [48]. Among the *Cutibacterium* genus, *C. acnes* (previously *Propionibacterium acnes*) is an important human pathogen. When the ability of *C. acnes* to remove Pb and Al from the intestine was studied in vitro, all four studied strains removed up to 57% of Pb and 24% of Al from the solution [49], providing evidence of the members of this genus being able to transform metals to insoluble form. The *Cutibacterium* spp. are also found as endophytes in grapevine [50] and *Crotalaria pumila* [51].

Interestingly, most of the bacterial taxa associated with Au-nanoparticles in spruce needles have earlier been found in the seeds of *C. pumila* as endophytes on a metal mining site with high concentrations of Zn, Pb, Cd, Cu, and Ni in the soil. When *C. pumila* was tested for phytoremediation, the bacterial communities of the seeds hosted the genera *Methylobacterium*, *Staphylococcus*, *Corynebacterium*, and *Cutibacterium*. Among these, the *Methylobacterium* genus was the most abundant one through three consecutive seed generations [51]. *Corynebacterium* has many well-known metal-resistant species, for example *C. glutamicum*, which is one of the most resistant microorganisms to arsenic [52] and can uptake Pd(II) as a biosorbent [53]. Similarly, many plant-associated *Methylobacterium* species can tolerate and accumulate high quantities of metals, such as Cu, Zn, and Ni [54–56]. All of these reports further support our findings.

However, to indisputably prove that Au is biomineralized by the specific bacterial species in Norway spruce needles would require further examination by future advanced technologies that have not been developed yet. For example, several practical challenges remain to be solved. The high Au concentration of needles and the presence of Au-nanoparticles did not always coincide with each other or with the tree location in the ore zone. Such discrepancy can be explained by the fact that biomineralization does not always take place, and when present, it can be sporadic and highly localized [57]. On the other hand, Au may occur in plant tissues as soluble complexes or in ionic forms that are not detectable by FE-SEM-EDS, which only visualizes precipitated, electron-dense particles. Advanced spectroscopic methods, such as X-ray absorption spectroscopy XAS or X-ray fluorescence spectroscopy XRF, are required to detect and characterize gold in these non-particulate states. To investigate endophytic bacterial communities in spruce needles, we employed 16S rRNA amplicon sequencing as an essential first step, recognizing its balance of accessibility and analytical value. While advanced methods, such as metagenomics and metabolomics are often recommended, their application to studying endophytes is limited by major technical challenges [58]. Bacterial endophytes closely resemble host mitochondria and chloroplasts, complicating their separation from host tissues using culture-independent methods without contamination [59]. High sequence similarity between bacterial and organellar rRNA genes, along with the lack of poly-A tails in bacterial transcripts, results in a predominance of host-derived sequences in RNA- and DNA based datasets [60]. While culturomics, as an advanced culture-dependent method, holds promise for future microbial characterization, a significant proportion of endophytic bacteria remain unculturable, especially in the shoot tissues of trees, due to their specific requirements for culture conditions, intimate association with host tissues, and slow growth. These issues contribute to the underrepresentation of endophyte studies, especially in non-model plants. To address this, we used peptide nucleic acid (PNA) PCR blockers [26] during 16S rRNA amplification to suppress amplification of host-derived sequences, targeting the DNA that encodes the 16S rRNA. This approach offered a more accurate overview of the endophytic community composition and provided a critical basis for identifying the candidate taxa for future functional analyses.

## Conclusions

We provided evidence supporting our hypothesis that endophytic bacteria are associated with biomineralization processes in plant tissue. We discovered Au-nanoparticles in the intercellular spaces of Norway spruce needle mesophyll covered by a microbial biofilm. The needles with high Au concentration showed a marked decrease in bacterial species richness. Specifically, an association of the bacterial vo *P3OB-42*, *Cutibacterium*,* Corynebacterium*, and *Methylobacterium* with the presence of the Au-nanoparticles was revealed. Members of these taxa have previously been found as endophytes in plants growing in metal-rich environments, supporting our results. In the future, further studies with advanced technologies are needed to elucidate the role of endophytic bacteria in Au biomineralization.

## Supplementary Information

Below is the link to the electronic supplementary material.

Supplementary Material 1

Supplementary Material 2

Supplementary Material 4

Supplementary Material 3

## Data Availability

No datasets were generated or analysed during the current study.
