# Supplementary figures and images for: Biomineralized gold nanoparticles along with endophytic bacterial taxa in needles of Norway spruce (Picea abies)
Source: Environ Microbiome. 2025 Aug 28;20:113. doi: 10.1186/s40793-025-00770-x (PMC12395814; doi:10.1186/s40793-025-00770-x)

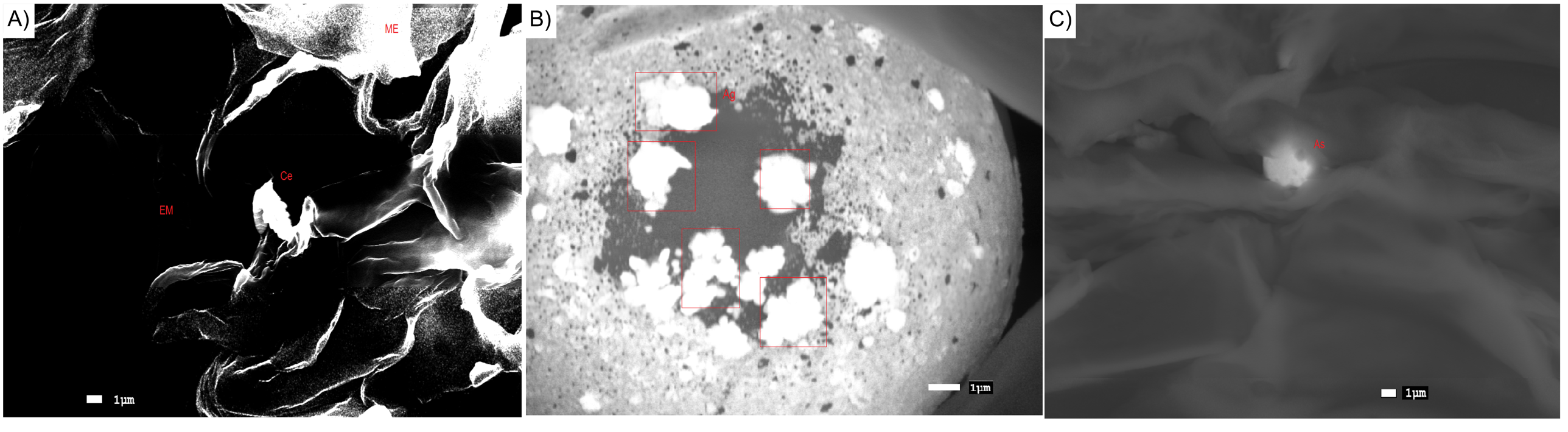

Supplement: Supplementary file 1 — Supplementary Material 1 [file 40793_2025_770_MOESM1_ESM.png]

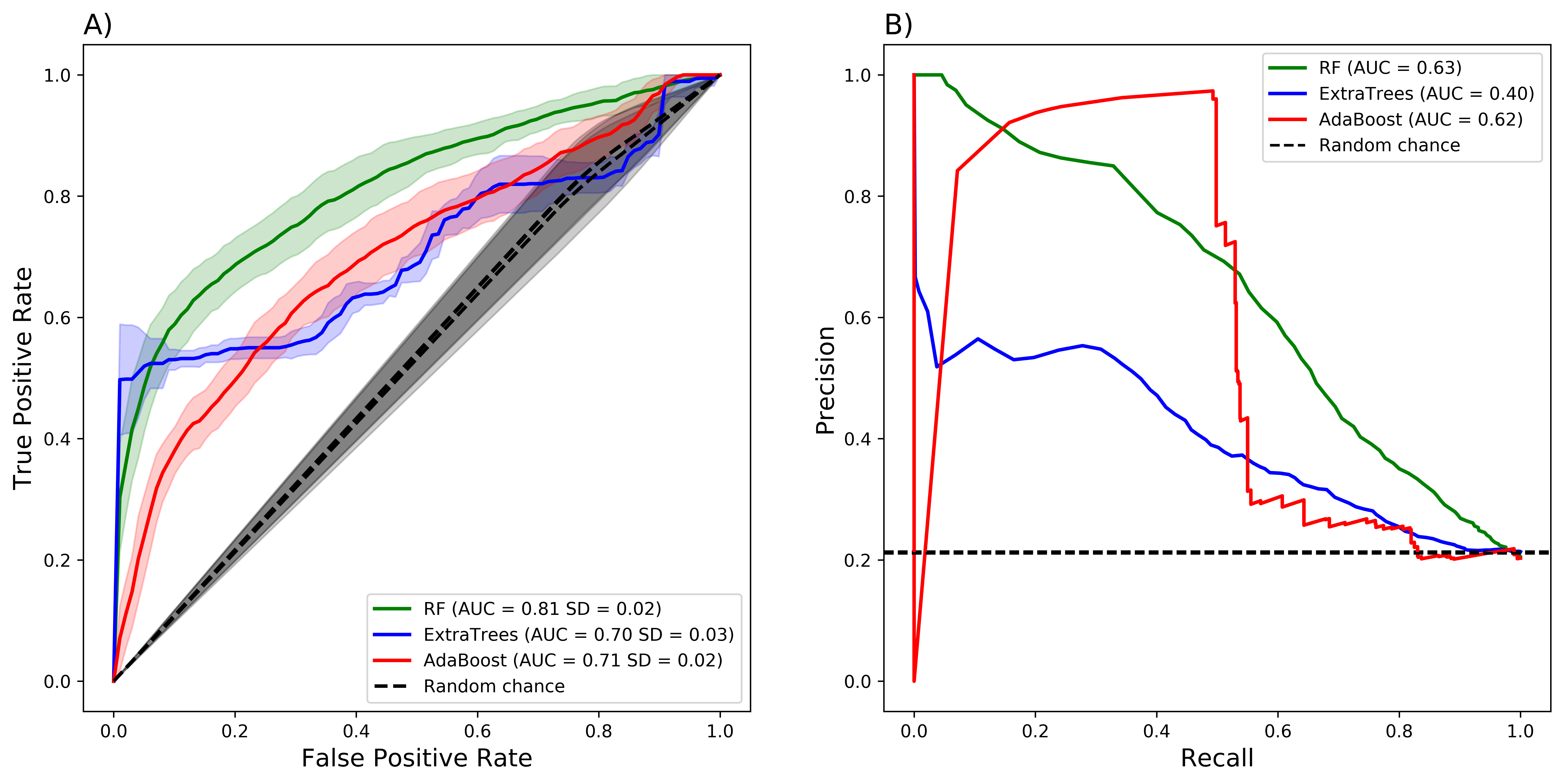

Supplement: Supplementary file 4 — Supplementary Material 3 [file 40793_2025_770_MOESM4_ESM.png]
